# Using siRNA to define functional interactions between melanopsin and multiple G Protein partners

**DOI:** 10.1007/s00018-014-1664-6

**Published:** 2014-06-24

**Authors:** Steven Hughes, Aarti Jagannath, Doron Hickey, Silvia Gatti, Matthew Wood, Stuart N. Peirson, Russell G. Foster, Mark W. Hankins

**Affiliations:** 1 Nuffield Laboratory of Ophthalmology, University of Oxford, Oxford, OX3 9DU UK; 2 F. Hoffman La Roche, RED Research and Development, CNS DTA, Basel, Switzerland; 3 Department of Anatomy, Physiology and Genetics, University of Oxford, Le Gros Clark Building, South Parks Road, Oxford, OX1 3QX UK

**Keywords:** Melanopsin, G protein, Phototransduction, Gene silencing, Pupilometry

## Abstract

Melanopsin expressing photosensitive retinal ganglion cells (pRGCs) represent a third class of ocular photoreceptors and mediate a range of non-image forming responses to light. Melanopsin is a G protein coupled receptor (GPCR) and existing data suggest that it employs a membrane bound signalling cascade involving Gnaq/11 type G proteins. However, to date the precise identity of the Gα subunits involved in melanopsin phototransduction remains poorly defined. Here we show that Gnaq, Gna11 and Gna14 are highly co-expressed in pRGCs of the mouse retina. Furthermore, using RNAi based gene silencing we show that melanopsin can signal via Gnaq, Gna11 or Gna14 in vitro, and demonstrate that multiple members of the Gnaq/11 subfamily, including Gna14 and at least Gnaq or Gna11, can participate in melanopsin phototransduction in vivo and contribute to the pupillary light responses of mice lacking rod and cone photoreceptors. This diversity of G protein interactions suggests additional complexity in the melanopsin phototransduction cascade and may provide a basis for generating the diversity of light responses observed from pRGC subtypes.

**Electronic supplementary material:**

The online version of this article (doi:10.1007/s00018-014-1664-6) contains supplementary material, which is available to authorized users.

## Introduction

Over the last decade it has become clear that photoreception within the mammalian retina is not restricted to the rod and cones of the outer retina [1, 2], but also extends to a small number of photosensitive retinal ganglion cells (pRGCs) expressing the blue light sensitive photopigment melanopsin (for review see [3–5]). These inner retinal photoreceptors provide information regarding environmental irradiance and mediate a range of non-image forming (NIF) responses to light including circadian entrainment, pupil constriction and the induction of sleep [1, 6–9], and may also contribute to visual pathways [10–12]. Since their original description, multiple distinct subtypes of pRGC have been identified (M1-M5 type pRGCs), characterised by differing levels of melanopsin expression and the stratification of their dendrites in specific sublamina of the inner plexiform layer (IPL) (for review see [4, 13]). In addition to these anatomical differences, a range of functional differences are now known to exist between these cell types, including the sensitivity and kinetics of photoresponses [10, 14–17], the nature of synaptic inputs received from the outer retina [18], and the isoforms of melanopsin that they express [19, 20]. These cell types also innervate different areas of the brain [16, 21–24] and mediate different physiological responses to light [24] (for review see [13, 25]).

The mechanisms of phototransduction in melanopsin expressing pRGCs are known to be markedly different from that of rod and cone photoreceptors, and more closely resemble an “invertebrate-like” phototransduction pathway (for review see [3–5, 26]). Stimulation of melanopsin leads to the activation of a membrane bound signalling cascade involving Gnaq/11 type G proteins, activation of phospholipase-C (PLC) and ultimately opening of downstream TRP type ion channels [27–31]. Recent studies have confirmed the precise identity of the PLC isoform involved, PLCβ4, and also the identity of the downstream ion channels, TRPC6 and TRPC7 [32]. However, despite the strong evidence that melanopsin couples to Gnaq/11 type G proteins within pRGCs [29, 31] and following expression in cell line systems [33–37], all previous studies have utilised pharmacological tools that fail to distinguish between the specific members of the Gnaq/11 family. As a result, the specific identity of the G protein sub-unit(s) involved in melanopsin phototransduction remains to be determined. The Gnaq/11 family contains four members, Gnaq, Gna11, Gna14 and Gna15 (Gna16 in humans) [38, 39]. Single cell PCR analysis indicates that mRNA for multiple members of this family can be detected within individual pRGCs (likely M1 type cells), with *Gna14* the most commonly detected [31]. However, to date the expression and localisation of Gnaq/11 type G proteins within the retina and within specific subclasses of pRGCs has not been investigated.

In this study we have used immunohistochemistry in combination with in vitro and in vivo siRNA based gene silencing techniques to determine the specific G protein Gα subunits with which melanopsin is capable of interacting. We conclude that melanopsin has multiple G protein partners available within pRGCs and is capable of signalling via Gnaq, Gna11 and Gna14 G proteins in vitro and in vivo.

## Results

### Expression of Gnaq/11 type G proteins in the mouse retina

**Fig. 1:**
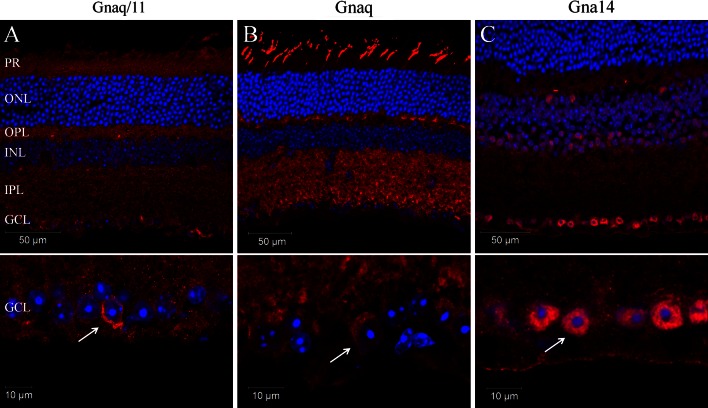
Localisation of Gnaq/11 type Gα subunits in the mouse retina. **a**–**c** Immunolabelling of the mouse retina with antibodies recognising both Gnaq and Gna11 (termed Gnaq/11) (**a**) or antibodies specific for Gnaq (**b**) or Gna14 (**c**) confirms the widespread expression of Gnaq/11 type G proteins in the wildtype mouse retina (upper panels) and specifically in cells of the ganglion cell layer (lower panels). For all images DAPI nuclear counter stain is shown in *blue*. *PR* Photoreceptors, *ONL* outer nuclear layer, *OPL* outer plexiform layer, *INL* inner nuclear layer, *IPL* inner plexiform layer, *GCL* ganglion cell layer. Additional images showing the detailed expression of Gnaq/11 type G proteins in the retina, and also data showing the specificity of these antibodies is shown in Supplementary Figs. 1 and 2

PCR and gene microarray analysis both show that mRNA transcripts for *Gnaq*, *Gna11* and *Gna14*, but not *Gna15* are expressed in the adult mouse retina (Supplementary Fig. 1). Given this profile of expression, we sought to determine the expression and localisation of Gnaq, Gna11 and Gna14 protein in the mouse retina and more specifically within melanopsin expressing pRGCs. However, due to the high sequence homology of Gnaq and Gna11 it is difficult to specifically label these Gα subunits with available antibodies. We have therefore performed immunostaining with antibodies raised against an epitope common to both Gnaq and Gna11 (termed Gnaq/11), or epitopes specific to Gnaq or Gna14 (Fig. 1; Supplementary Fig. 1). The specificity of the Gnaq/11, Gnaq and Gna14 antibodies was confirmed by their ability to label Neuro-2A cells transiently transfected with plasmids encoding their target proteins (Supplementary Fig. 1b). Labelling was also performed with an antibody that recognises four of the five Gβ subunits (Gβ1, 2, 3 and 4 but not Gβ5) (Fig. 1; Supplementary Fig. 1c).

Consistent with the ubiquitous expression of Gnaq and Gna11, labelling with the Gnaq/11 antibody showed a wide spread pattern of expression within the retina (Fig. 1a). Gnaq/11 labelling was observed in all layers of the retina, including the photoreceptor layer (PR), the outer plexiform layer (OPL), inner nuclear layer (INL), inner plexiform layer (IPL) and ganglion cell layer (GCL). Interestingly, a distinct increase in Gnaq/11 labelling was observed for a small subset of cells located within the GCL and to a lesser extent the INL. Labelling with the Gnaq antibody showed a similar widespread pattern of expression to that observed with the Gnaq/11 antibody (Fig. 1b). However, the Gnaq antibody labelled only cone photoreceptors in the outer retina and produced a more uniform staining of cells in the GCL. Compared to Gnaq and Gna11, the expression of Gna14 in the retina is more restricted (Fig. 1c). The highest levels of Gna14 immunoreactivity were observed in the GCL, where approximately 70 % of cells showed strong intracellular labelling. Low levels of labelling were also observed in the IPL and INL, whereas labelling was entirely absent from the outer nuclear layer (ONL) and photoreceptor layer. Similarly to Gnaq/11 labelling, distinctive membrane labelling of Gna14 was detected for a small number of cells located in both the GCL and INL. Labelling with an antibody that recognises Gβ subunits (a necessary component of G protein signalling complexes) showed a similar pattern of expression to that observed for Gnaq/11 and Gnaq antibodies, albeit with much increased expression of Gβ detected in the photoreceptor layer (consistent with high expression of rhodopsin and cone opsin GPCRs). In all cases, omission of primary antibodies resulted in no appreciable staining. Additional images showing the detailed distribution of Gnaq/11 type G proteins in the mouse retina, including co-labelling of Gnaq/11 and Gnaq antibodies, are shown in Supplementary Fig. 1 and 2.

### Melanopsin expressing retinal ganglion cells express Gnaq, Gna11 and Gna14 G protein α subunits

**Fig. 2:**
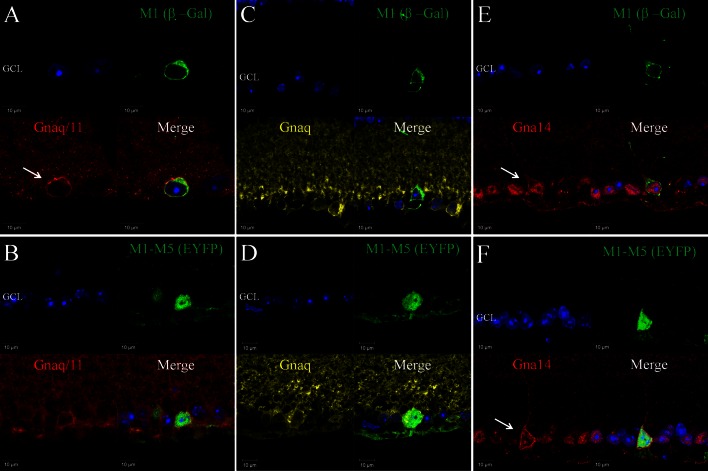
Expression of Gnaq, Gna11 and Gn14 in pRGC subtypes of the mouse retina. Double labelling of G protein antibodies and markers of specific pRGC subtypes shows the widespread expression of Gnaq, Gna11 and Gna14 in all pRGC subtypes. **a**, **b** Gnaq/11 immunoreactivity (*red*) was consistently detected for β-Gal positive M1 cells (*green*) (**a**) and EYFP positive M1-M5 cells (*green*) (**b**), although levels of membrane bound Gnaq/11 labelling are increased for M1 type pRGCs. **c**, **d** Gnaq immunoreactivity (*yellow*) was consistently detected for β-Gal positive M1 cells (*green*) (**c**) and EYFP positive M1-M5 cells (*green*) (**d**). The levels and pattern of Gnaq labelling were similar for β-Gal positive M1 cells, EYFP positive M1-M5 cells and neighbouring non-melanopsin cells. **e**, **f** Gna14 immunoreactivity (*red*) was consistently detected for β-Gal positive M1 cells (*green*) (**e**) and EYFP positive M1-M5 cells (*green*) (**f**). Membrane-bound Gna14 labelling was typically observed for M1 type pRGCs (**e**, *white arrow*), whereas only intracellular Gna14 labelling was detected for the majority of EYFP positive cells and neighbouring non-melanopsin cells. Membrane bound Gna14 labelling was observed for a subset of EYFP cells resembling M1 type pRGCs (**f**, *white arrow*). For all images DAPI nuclear counter stain is shown in *blue*. GCL is ganglion cell layer

**Fig. 3:**
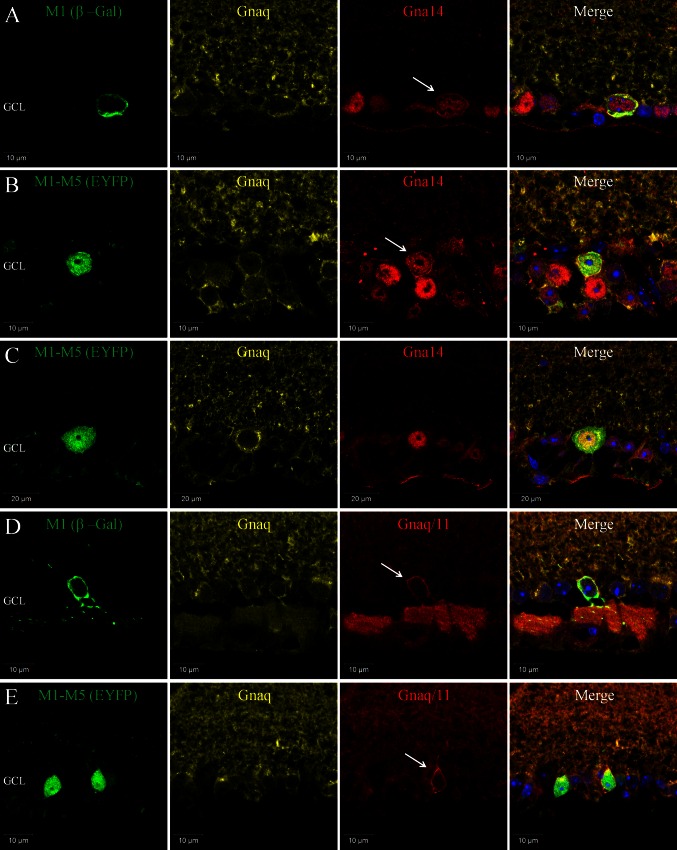
Co-expression of Gnaq, Gna11 and Gn14 in subtypes of pRGC. **a** Triple labelling of β-Gal (*green*), Gna14 (*red*) and Gnaq (*yellow*) antibodies confirms the co-expression of Gna14 and Gnaq in M1 type pRGCs. Levels and localisation of Gnaq labelling are similar between M1 cells and neighbouring non-melanopsin cells, whereas only M1 type cells show membrane-bound and intracellular labelling of Gna14 (white arrow). **b**, **c** Triple labelling of EYFP (*green*), Gna14 (*red*) and Gnaq (*yellow*) confirms the co-expression of Gna14 and Gnaq in M1-M5 type pRGCs. Expression of Gna14 is detected in all melanopsin cells, with distinctive membrane labelling of Gna14 evident for a subset of EYFP cells (*white arrow*), consistent with the percentage of M1 type pRGCs. The levels and distribution of Gnaq labelling show no obvious change within individual pRGCs regardless of their pattern of Gna14 labelling. **d** Triple labelling of β-Gal (*green*), Gnaq/11 (*red*) and Gnaq (*yellow*) antibodies confirms the co-expression of Gnaq and Gna11 Gα subunits in M1 type pRGCs. Levels of Gnaq labelling are similar between M1 cells and neighbouring non-melanopsin cells. By contrast, M1 cells show increased membrane bound Gnaq/11 labelling (*white arrow*), consistent with increased expression of Gna11 in these cells. **e** Triple labelling of EYFP (*green*), Gnaq/11 (*red*) and Gnaq (*yellow*) antibodies confirms the co-expression of Gnaq and Gna11 in M1-M5 type pRGCs. Both Gnaq/11 and Gnaq antibodies label all melanopsin cells, although increased levels of Gnaq/11 labelling are evident for only a subset of EYFP cells, with morphologies characteristic of M1 type pRGCs (*white arrow*)

To enable localisation of Gnaq, Gna11and Gna14 Gα subunits within specific subtypes of melanopsin expressing pRGCs, double and triple labelling was performed on retina sections from heterozygous *Opn4* ^+*/*−^(*tau*-*LacZ* ^+*/*−^) mice that express a β-Gal reporter selectively within M1 type pRGCs [21, 40] and also retina from *Opn4* ^+*/*−^Cre^+*/*−^EYFP mice in which an EYFP reporter is expressed in M1-M5 type pRGCs [10, 41] (Figs. 2, 3). Collectively our data are consistent with the widespread expression of Gnaq, Gna11 and Gna14 within all subtypes of pRGC present in the mouse retina, including M1-M5 type pRGCs, although levels of Gna11 and the cellular localisation of Gna14 would seem to vary between pRGC subtypes.

Gnaq/11, Gnaq and Gna14 antibodies all consistently stained β-gal positive M1 cells and also EYFP positive M1-M5 type pRGCs (Fig. 2). Gnaq/11 immunoreactivity was detected for 100 % of β-gal positive M1 cells and 100 % of EYFP positive M1-M5 cells (*n* = 52 and *n* = 102 cells counted, respectively) (Fig. 2a, b). Interestingly, levels of membrane bound Gnaq/11 immunoreactivity were increased for the majority of β-gal positive M1 cells (76.9 %, *n* = 52) compared to non-M1 cells and other neighbouring cells in the ganglion cell layer (Fig. 2a). A similar increase in Gnaq/11 labelling was also observed for a subset of EYFP cells (17.6 %, *n* = 102 cells), corresponding to the expected proportion of M1 type pRGCs [41]. This distinctive pattern of Gnaq/11 labelling was only rarely detected for non-melanopsin cells. Similarly to the Gnaq/11 antibody, Gnaq immunoreactivity was also detected for 100 % of β-gal and EYFP positive pRGCs (*n* = 46 and *n* = 78 cells counted, respectively) (Fig. 2c, d). However, by contrast to labelling with the Gnaq/11 antibody, there were no observable differences in levels or pattern of Gnaq labelling observed between β-gal and EYFP positive cells, or when compared to other cells located in the ganglion cell layer. Based on the specificity of the Gnaq/11 and Gnaq antibodies (Supplementary Fig. 1) it is likely that the increased Gnaq/11 immunoreactivity observed for M1 type pRGCs is due to increased expression of Gna11 in these cells (see also Supplementary Fig. 2).

Labelling with the Gna14 antibody indicates the widespread expression of Gna14 in close to 100 % of all pRGCs, including M1-M5 type pRGCs. Gna14 immunoreactivity was detected in 89.7 % (*n* = 58) of β-gal positive M1 cells and 94.7 % (*n* = 113) of EYFP positive M1-M5 cells (Fig. 2e, f). For the majority of EYFP positive cells, only intracellular labelling of Gna14 was readily detected (83.2 %, *n* = 113), similar to that detected for non-melanopsin cells in the GCL. However, similar to Gnaq/11, distinctive labelling of Gna14 was detected at the cell membrane for a subset of melanopsin cells, with this pattern of labelling again restricted to M1 type pRGCs. Clear membrane bound expression of Gna14 was detected for 70.7 % (*n* = 58) of β-gal positive M1 cells (Fig. 2e), including displaced M1 type pRGCs, and 16.8 % (*n* = 113) of EYFP cells (Fig. 2f). In all cases where the morphology of cells was clearly evident, EYFP cells with distinctive membrane-bound expression of Gna14 resembled M1 type cells with processes extending towards the surface of the inner plexiform layer (IPL). A summary of G protein expression within subsets of pRGCs is shown in Table 1.

**Table 1:** Summary of Gnaq/11 type G protein expression in pRGC subtypes as determined by antibody labelling of retinal sections

	% ir-β-gal cells (M1 pRGCs)	% ir-β-gal cells membrane labelling	% ir-EYFP cells (M1-M5 pRGCs)	% ir-EYFP cells membrane labelling
Gnaq/11	100 (*n* = 52)	77.0 (*n* = 52)	100 (*n* = 102)	17.6 (*n* = 102)
Gnaq	100 (*n* = 46)	0 (*n* = 46)	100 (*n* = 78)	0 (*n* = 78)
Gna14	89.7 (*n* = 58)	70.7 (*n* = 58)	94.7 (*n* = 113)	16.8 (*n* = 113)

Data show the percentage of β-gal positive M1 type pRGCs and EYFP positive M1-M5 type pRGCs that were positively labelled with Gnaq/11, Gnaq and Gna14 antibodies. The percentage of cells for which distinctive membrane bound labelling was observed is also shown. *n* refers to the number of individual cells analysed

### Gnaq, Gna11 and Gna14 G proteins are highly co-expressed in pRGCs

Based on the percentage of β-gal and EYFP cells for which Gnaq/11, Gnaq or Gna14 immunoreactivity are detected, it is clear that individual pRGCs likely co-express multiple members of the Gnaq/11 sub-family of G proteins. Triple labelling for β-gal (M1 pRGCs) or EYFP (M1-M5 pRGCs) and Gnaq and Gna14 confirms the widespread co-expression of these Gα subunits within individual pRGCs (Fig. 3). Gnaq and Gna14 are co-expressed in close to 100 % of all individual pRGCs examined, including β-gal positive M1 type pRGCs (96 %, *n* = 50 cells) (Fig. 3a) and EYFP positive M1-M5 type pRGCs (96.3 %, *n* = 107 cells) (Fig. 3b, c). Membrane bound Gna14 labelling is again detected for the majority of β-gal positive M1 cells (80 %, *n* = 50 cells) (Fig. 3a) and a subset of EYFP cells approximately corresponding to the proportion of M1 cells (16.8 %, *n* = 107 cells) (Fig. 3b). The levels and distribution of Gnaq labelling show no obvious change within individual pRGCs regardless of their pattern of Gna14 labelling. High levels of co-expression were also detected when performing triple labelling with Gnaq/11 and Gnaq antibodies. Increased membrane labelling of Gnaq/11 but not Gnaq was again observed for β-gal positive M1 type pRGCs (Fig. 3d) and a subset of EYFP cells (Fig. 3e) consistent with increased levels of Gna11 expression in M1 type pRGCs. Due to species limitations it was not possible to perform triple labelling with Gnaq/11 and Gna14 antibodies. However, based on the levels of expression observed for Gnaq, Gna11 and Gna14, it is clear that the majority of individual pRGCs likely co-express all three of these G proteins, but the levels of Gna11 and Gna14 detected at the cell membrane are seemingly increased for M1 type pRGCs.

### Using siRNA to define melanopsin G protein interactions in vitro

Heterologous expression of melanopsin has been show to confer photosensitivity to a range of cell lines [33, 35], typically resulting in the activation of a classical Gnaq/11 type G protein signalling pathway and release of calcium from intracellular stores [34, 36, 37, 42]. We have used a similar in vitro model, in combination with gene silencing and gene over expression techniques to investigate the specific identity of the Gα subunits with which melanopsin is capable of interacting. For this purpose we first generated Neuro2A cell lines that stably express either the long or short isoforms of mouse melanopsin (Opn4L or Opn4S) [19] and confirmed that these cell lines show light induced calcium responses consistent with the expression of melanopsin. For a detailed characterisation of the Opn4L and Opn4S expressing Neuro-2A cell lines see Supplementary Fig. 3.

**Fig. 4:**
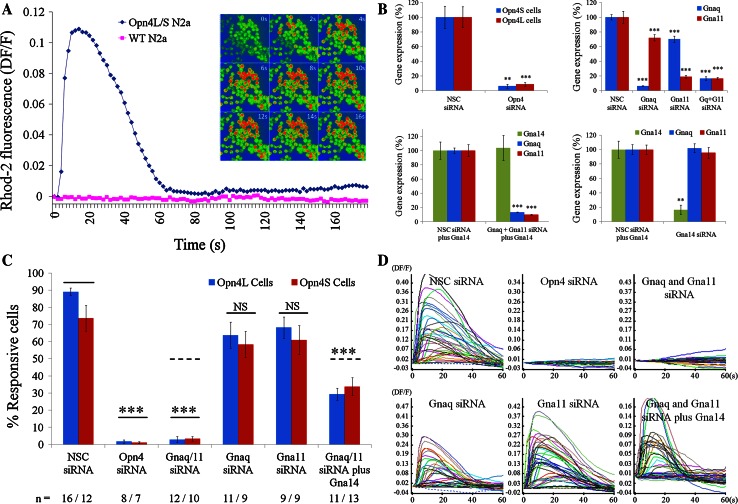
Melanopsin can couple to Gnaq, Gna11 and Gna14 in vitro. An in vitro cell line model of melanopsin signalling was used in combination with gene silencing and gene over expression techniques to show that melanopsin can couple to Gnaq, Gna11 and Gna14 Gα subunits. **a** Opn4L and Opn4S expressing Neuro-2A cells but not wildtype (WT) Neuro-2A cells show rapid and robust changes in intracellular calcium levels following the onset of image acquisition and resulting light stimulation (Rhod-2 images collected every 2 s using 100 ms exposures to 545 nm light at 7.9 × 10^13^ photons cm^−2^ s^−1^,10 nm bandwidth). Traces shown are the mean of all cells imaged in a single experiment (typically ~60–80 cells). Time lapse images (0–16 s) show rapid changes in intracellular calcium upon light exposure, with peak elevations typically observed within 10–20 s. **b** Graphs showing levels of mRNA expression observed following transfection of Neuro-2A cells with siRNA in vitro. Opn4 siRNA reduced expression of both isoforms of melanopsin, *Opn4L* and *Opn4S,* by 90–95 % (top left). Gnaq and Gna11 siRNA selectively silence *Gnaq* or *Gna11* mRNA by 90 and 85 %, respectively (top right). Co-transfection of Gnaq and Gna11 siRNA resulted in 80–90 % silencing for both genes (top right). Co-silencing of *Gnaq* and *Gna11* was also achieved following simultaneous transfection with plasmid DNA encoding Gna14 (lower left). Gna14 siRNA silenced *Gna14* mRNA by 85–90 % (lower right). All gene expression data are normalised to the geometric mean expression of three housekeeping genes (*Gapdh, Arbp*, *β-actin*) and shown as percentage expression compared to cells transfected with non-sequence control (NSC) siRNA. *n* = 5 replicate cultures for all groups. Data are shown as mean ± SEM. ** *t* test *p* < 0.01, *** *t* test *p* < 0.001. **c**, **d** Combining calcium imaging and siRNA transfection shows that both the long and short isoforms of melanopsin, Opn4L and Opn4S, can couple to Gnaq, Gna11 and Gna14 in vitro. **c** Graph showing the percentage of Opn4L and Opn4S expressing cells that show light induced changes in calcium levels following transfection with siRNA. Responses are eliminated following silencing of Opn4, and following co-silencing of Gnaq and Gna11. Robust responses are observed for cells expressing only Gnaq (receiving Gna11 siRNA), cells expressing only Gna11 (Gnaq siRNA) and cells expressing only Gna14 (Gnaq and Gna11 siRNA plus Gna14 plasmid). The number of replicate experiments (dishes) performed for each set of conditions is shown below the main graph. *** *t* test *p* < 0.001 following Bonferroni multiple test correction. **d** Traces showing responses recorded from melanopsin expressing cells receiving the different combinations of siRNA and plasmid DNA. Panels show responses from all individual cells within a single experiment (typically 50–60 cells)

For both Opn4L and Opn4S expressing cells, but not wild-type (WT) Neuro-2A cells, changes in intracellular calcium levels were evident following light stimulation, with the majority of cells (typically ~80–90 %) showing rapid and robust changes in intracellular calcium levels in response to the onset of the imaging protocols (and exposure to the light stimuli used to acquire the Rhod-2 fluorescence images) (Fig. 4a). Elevations in intracellular calcium levels were transient with levels of fluorescence typically returning to baseline within 60–90 s. These responses were absent from cells not loaded with 9-*cis* retinal chromophore (0 % responsive cells, *n* = 3 dishes) and were abolished following depletion of intracellular calcium stores with 1 μM thapsigargin (0 % responsive cells, *n* = 3 dishes). Based on these data, it is likely that the observed responses stem from the activation of a classical Gnaq/11 type signalling pathway involving the generation of IP_3_ and the release of calcium from internal stores.

Based on qPCR analysis, Neuro-2A cells natively express high levels of Gnaq and Gna11, but not Gna14 or Gna15 (Supplementary Fig. 3). In order to investigate the contribution of Gnaq and Gna11 to the melanopsin responses observed from Neuro-2A cells we utilised siRNA to silence selectively the expression of these Gα subunits. For each gene of interest, levels of mRNA silencing in the region of 85–95 % were achieved (Fig. 4b). Co-transfection of Neuro-2A cells with siRNA targeting both Gnaq and Gna11 resulted in significant levels of co-silencing for both genes (~80–90 %) and co-silencing of both Gnaq and Gna11 was also achieved following simultaneous transfection with plasmid DNA encoding Gna14. Immunostaining of Neuro-2A cells transiently transfected with plasmid DNA and the corresponding siRNA showed similar levels of protein silencing, with target proteins detected at only minimal levels compared to cells receiving plasmid DNA and NSC siRNA (Supplementary Fig. 4). In summary, using the selected siRNA sequences it was possible to generate melanopsin expressing Neuro-2A cells that express Gnaq and Gna11, or Gnaq, Gna11 or Gna14 alone, or cells that express only minimal levels of any of these Gα subunits. We also validated siRNA sequences targeting Opn4 (to act as a positive control) and Gna14 (for latter in vivo use).

Transfection of Opn4L and Opn4S expressing Neuro-2A cells with siRNA targeting Opn4 led to the elimination of light response in these cells (>95 % reduction in the number of responsive cells compared to cells transfected with NSC siRNA, *t* test *p* < 1.1E−5 for both Opn4L and Opn4S cells) (Fig. 4c, d). Co-transfection of siRNA targeting both Gnaq and Gna11 also resulted in a complete elimination of the melanopsin driven responses in both Opn4L and Opn4S expressing cells (>95 % reduction, *t* test *p* < 1.2E−5 for both Opn4L and Opn4S cells). Overall the effect of co-silencing both Gnaq and Gna11 was similar to that observed following the silencing of Opn4. These results suggest, as expected, that the melanopsin driven responses in these cells are acting via the Gnaq/11 signalling pathway. By contrast to Gnaq and Gna11 co-silencing, robust responses were clearly evident from both Opn4L and Opn4S expressing cells when either Gnaq or Gna11 was silenced alone. The nature of responses observed (amplitude, kinetics and percentage of responsive cells) was comparable in cells treated with either Gnaq or Gna11 siRNA alone (express only Gna11or Gnaq) and was not significantly different from cells treated with NSC siRNA (express both Gnaq and Gna11). Examples of responses recorded from each group of cells are shown in Fig. 4d). Collectively these results suggest that both isoforms of mouse melanopsin, Opn4L and Opn4S, are capable of signalling via either Gnaq or Gna11, and it is necessary to silence both Gnaq and Gna11 in order to entirely uncouple the melanopsin signalling pathway in Neuro-2A cells.

To assess whether melanopsin can also couple to Gna14 this gene was transiently transfected into Opn4L and Opn4S expressing cells in which both Gnaq and Gna11 were simultaneously co-silenced. Under these conditions the expression of Gna14 was sufficient to rescue melanopsin dependent responses in ~40 % of cells (*t* test *p* < 0.0001 for both Opn4L and Opn4S cells) (Fig. 4c), consistent with levels of transient Gna14 plasmid transfection observed under these conditions (Supplementary Fig. 1b). The properties of these responses were broadly similar to those observed in the presence of Gnaq and or Gna11 (Fig. 4d). These data suggest that both Opn4L and Opn4S are capable of coupling to Gna14 in the absence of Gnaq and Gna11. In summary, melanopsin dependent responses were observed in Neuro-2A cells expressing Gnaq, Gna11 or Gna14 in isolation and were completely abolished in cells lacking all three Gα subunits. Based on these results, both isoforms of mouse melanopsin appear capable of coupling to Gnaq, Gna11 and Gna14 in vitro.

### Simultaneous silencing of Gnaq, Gna11 and Gna14 is required to attenuate melanopsin driven pupillary light responses in vivo

**Fig. 5:**
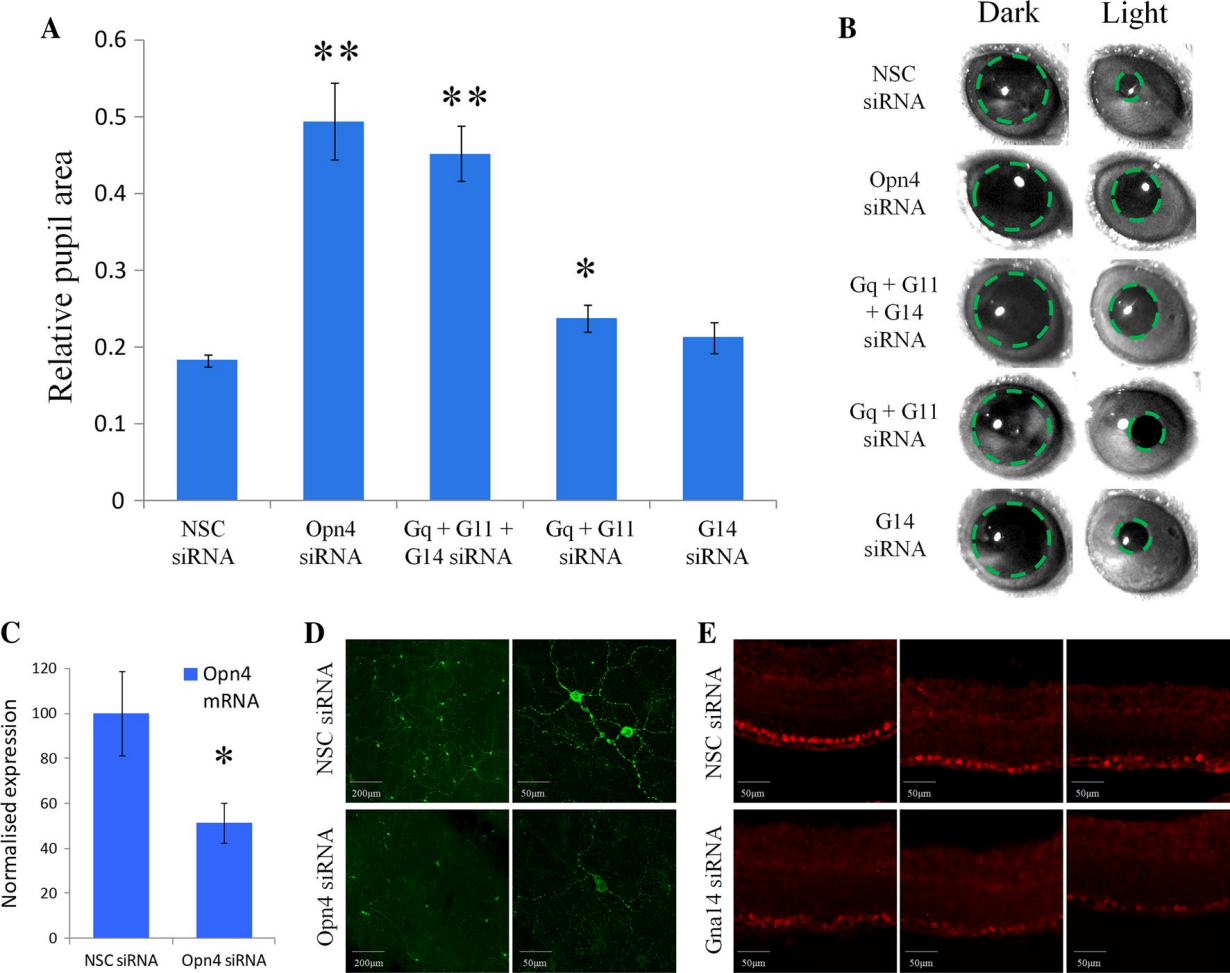
Gnaq/11 and Gna14 G proteins perform overlapping roles in melanopsin phototransduction in vivo. **a** Summary of pupillary light responses observed from mice receiving intravitreal injections of siRNA targeting Opn4, Gna14, both Gnaq and Gna11, Gnaq and Gna11 and Gna14, or non-sequence control (NSC) siRNA. A significant attenuation of the pupil light response was observed following silencing of Opn4 and also following the simultaneous silencing of Gnaq, Gna11 and Gna14. A small, but significant reduction in pupil constriction was also observed following co-silencing of Gnaq and Gna11, but not following the silencing of Gna14 alone. Data are shown as mean ± SEM. * = *t* test *p* < 0.02, ** = *t* test *p* < 0.002. **b** Images showing pupil area immediately before the onset and termination of light stimulation for mice receiving the various siRNA. **c** Levels of *Opn4* mRNA detected in the mouse retina following delivery of NSC siRNA and Opn4 siRNA. Data is shown as mean ± SEM. * = *t* test *p* = 0.035. **d** Images showing levels of melanopsin protein detected in whole retina flatmounts from mice 72 h post injection of NSC siRNA and Opn4 siRNA. **e** Images showing levels of Gna14 protein detected 72 h post injection of NSC siRNA and Gna14 siRNA. For both Opn4 and Gna14 siRNA notable but incomplete levels of protein silencing are observed

Following the confirmation that melanopsin can couple to Gnaq, Gna11 and Gna14 in vitro we next sought to determine the role of these Gα subunits in melanopsin phototransduction in vivo. In the absence of rod and cone photoreceptors the pupillary light response is driven exclusively by melanopsin expressing pRGCs [7, 43–45]. For this reason, the pupillary light response of *rd/rd cl* mice lacking rod and cone photoreceptors was used as an in vivo assay of melanopsin function (Fig. 5).

Intravitreal injection of anti-melanopsin siRNA resulted in a significant attenuation of the pupillary light response compared to treatment with NSC siRNA (Fig. 5a, b). Following in vivo silencing of melanopsin, pupil area was reduced to 49.4 ± 5.0 % (*n* = 3 animals) of dark adapted values (~50 % constriction) following light stimuli, as compared to 18.2 ± 0.8 % (*n* = 5) observed for NSC control treated animals (~80 % constriction) (*t* test *p* = 0.0002). By contrast, pupillary light responses were not significantly influenced by silencing of Gna14 alone (*n* = 4, *t* test *p* = 0.17), and showed only a small reduction following silencing of both Gnaq and Gna11 (*n* = 4, *t* test *p* = 0.02). However, pupillary light responses were significantly attenuated following the simultaneously silencing of all three Gα subunits, Gnaq, Gna11 and Gna14, with pupil area reduced to 45.2 ± 3.6 % (*n* = 7) of dark adapted values, compared to 18.2 ± 0.8 % (*n* = 5) for NSC siRNA (*t* test *p* = 0.0001). Under these conditions the levels of pupil constriction were comparable to that observed following silencing of melanopsin itself. These results suggest an overlapping role for Gnaq/Gna11 and Gna14 Gα subunits in melanopsin signalling in vivo and indicate that silencing of Gnaq/Gna11 and Gna14 is necessary to suppress melanopsin phototransduction and the ability of pRGCs to drive the pupilliary light response.

Although we observe a clear attenuation of the melanopsin-driven pupillary light response from degenerate *rd/rd cl* mice following in vivo delivery of Opn4 siRNA, and also following the combined delivery of Gnaq, Gna11 and Gna14 siRNA, the resulting levels of pupil constriction remain significantly higher than those observed from *Gnat* ^−*/*−^, *Cnga3* ^−*/*−^, *Opn4* ^−*/*−^ triple knockout mice in which the function of all classes of photoreceptors are completely disrupted and show no measurable pupil constriction [46]. These observations would indicate an incomplete silencing of target proteins in vivo in our studies. In agreement with this conclusion, analysis of both mRNA and protein expression indicates an incomplete silencing of target proteins following in vivo delivery of siRNA. qPCR analysis shows that levels of *Opn4* mRNA are reduced by 48.5 ± 8.9 % (*n* = 8, *p* = 0.035) 72 h following intravitreal injection of Opn4 siRNA (Fig. 5). These data confirm the silencing of target mRNA within pRGCs of the degenerate mouse retina in vivo. Consistent with levels of mRNA silencing, levels of protein silencing were typically in the region of 40–70 % (assessed 72–96 h post injection). Examples of the levels of protein silencing typically observed for melanopsin and Gna14 siRNA are shown in Fig. 5c, d (see also Supplementary Fig. 5).

## Discussion

There is a growing acceptance that melanopsin signals via interactions with Gnaq/11 type G proteins. However, to date the precise identity of the Gα subunits involved in melanopsin phototransduction remains to be determined. To that end, this study provides the first detailed investigation of melanopsin G protein interactions. Our data confirm the expression and localisation of Gnaq, Gna11 and Gna14 G proteins in the mouse retina and more specifically within melanopsin expressing retinal ganglion cells, including M1-M5 type pRGCs. Furthermore, we have used a combination of in vitro and in vivo siRNA to determine the specific G protein Gα subunits with which melanopsin is capable of interacting.

Our data show that Gnaq, Gna11 and Gna14 proteins are highly co-expressed within all pRGC subtypes of the mouse retina. Based on the levels of co-expression observed for Gnaq, Gna11 and Gna14 with markers of specific pRGC subtypes, it would seem likely that all pRGC subtypes, and the majority of all individual pRGCs co-express at least three members of the Gnaq/11 family of G proteins, including Gnaq, Gna11 and Gna14. There are however a number of key differences observed between different pRGC subtypes. Levels of Gnaq expression show no obvious variations between pRGC subtypes or other cells in the ganglion cell layer. By contrast M1 type pRGCs show higher levels of Gna11 (deduced by comparing the pattern of labelling observed for Gnaq and Gnaq/11 antibodies) and Gna14 expression at the cell membrane. The increase in membrane bound G protein expression observed for M1 cells may be due to the high levels of melanopsin expression in these cells. However, the fact that Gna11 and Gna14, but not Gnaq show distinctive localisation at the cell membrane of M1 cells may suggest that these G proteins are the preferred partners for melanopsin within M1 type pRGCs, where all components of the phototransduction cascade have been shown to be included in membrane bound signalling complexes [31].

The long and short isoforms of mouse melanopsin, Opn4L and Opn4S, are differentially expressed in pRGC subtypes; M1 cells express both Opn4L and Opn4S, whereas only Opn4L is detected in M2 cells [19, 20]. Using an in vitro cell line model of melanopsin signalling we demonstrate that both isoforms of mouse melanopsin, Opn4L and Opn4S, are capable of coupling to Gnaq, Gna11 and Gna14 G protein partners in vitro. Results obtained following co-silencing of Gnaq and Gna11 were identical to those observed following silencing of melanopsin itself, with a near complete elimination of light responses observed for Opn4L and Opn4S expressing cell lines. These data confirm that Gnaq and Gna11 convey the melanopsin signal in Neuro-2A cells. Subsequent experiments demonstrated that both Opn4L and Opn4S are capable of signalling via either Gnaq or Gna11, as silencing of both genes was necessary to eliminate melanopsin dependent responses in these cells. Furthermore, the addition of Gna14 was sufficient to rescue responses in cells in which both Gnaq and Gna11 were silenced. There were no detectable differences observed between Opn4L and Opn4S expressing cells under any conditions tested and we conclude that both isoforms of mouse melanopsin are capable of signalling via Gnaq, Gna11 and Gna14 in vitro. The epitopes involved in G protein interactions are thought to reside in the intracellular loops of 7-transmembrane spanning GPCRs [47–49] and as the long and short isoforms of mouse melanopsin differ only in their distal C-terminal tails [19] it is perhaps not surprising that we find no differences in the profiles of G proteins with which these isoforms interact.

In addition to these in vitro studies, our data also confirm a diversity of melanopsin G protein interactions in vivo. Using intravitreal injections of siRNA we demonstrate an overlapping role for Gnaq, Gna11 and Gna14 in melanopsin driven pupillary light responses. Pupillary light responses were largely unaltered in mice receiving a combination of Gnaq and Gna11 siRNA or Gna14 siRNA alone, whereas simultaneous silencing of all three Gα subunits resulted in a significant attenuation of the pupil light response in a manner comparable to that seen following silencing of melanopsin. Given that silencing Gna14 alone did not result in a deficit in pupil responses we can conclude that melanopsin is capable of coupling to either Gnaq or Gna11 in vivo. Given the complete functional overlap of these Gα subunits [50, 51] it would seem highly likely that melanopsin is capable of coupling to both. Similarly, the lack of a notable deficit following co-silencing of Gnaq and Gna11 indicates that melanopsin can also couple to Gna14. Collectively our results show that melanopsin may couple to either Gnaq, Gna11 or Gna14, or a combination of all three. It is possible that this variability of G protein partners may offer some functional diversity to the melanopsin signalling pathway and may provide a basis for generating the diversity of light responses observed from individual pRGCs [27, 52].

Due to the high degree of sequence homology (90 %) and the known functional overlap of Gnaq and Gna11, the finding that melanopsin is capable of signalling via either of these sub-units is not unexpected. The finding that melanopsin can also couple to Gna14 is more noteworthy. Unlike Gnaq and Gna11, expression of Gna14 is largely restricted to a small number of specialised cell types [53–56], where presumably it imparts specific functions that are not shared by Gnaq or Gna11. Unfortunately, the functional differences between Gnaq/Gna11 and Gna14 are not well characterised. The profile and efficiencies with which Gnaq, Gna11 and Gna14 activate PLC-β isoforms (the major effector enzyme of Gnaq/11 type G proteins) are very similar. All three subunits are potent activators of PLC-β1, β3 and β4, and all show relatively weak activation of PLC-β2 (for review see [51]). However, Gnaq/11 type G proteins are involved in a number of cell signalling events that are independent of PLC activation [51], and thus functional differences may reside in these secondary signalling events. Although there is a high degree of overall sequence homology between members of the Gnaq/11 family (Gna11 and Gna14 share a 90 and 80 % overall sequence homology with Gnaq), the N-terminal regions of these G proteins are far more diverse (65 % identity between the first 40 amino acids of Gnaq and Gna14). The N-terminal regions of Gα subunits contain numerous sites targeted by post translational modifications, and perform important roles in cellular trafficking, membrane attachment and protein–protein interactions, including the binding of G protein β subunits (known signalling mediators) and scaffold type proteins which aid in the assembly of localised signalling complexes [50, 51]. Thus variability in the N-terminal domains may provide a mechanism for generating functional diversity between Gnaq/Gna11 and Gna14 based signalling events (for example see [57]).

In summary, this study provides the first detailed investigation of melanopsin G protein interactions. We show that Gnaq, Gna11 and Gna14 are available within all pRGC subtypes, and furthermore we show that both the long and short isoforms of mouse melanopsin are capable of coupling with Gnaq, Gna11 and Gna14 in vitro and in vivo. Based on these data, melanopsin seems to be capable of interacting with multiple G protein partners. These findings represent a new level of complexity in the melanopsin phototransduction cascade and may provide a basis for generating the diversity of light responses observed from individual pRGCs [27, 52]. Furthermore, this study highlights the advantages of using siRNA based approaches to identify the precise components of cell signalling cascades, with siRNA offering levels of target specificity not available with traditional pharmacology.

## Materials and methods

### Animals

Several strains of mice were utilised in this study. Wild type mice C3H/He mice (not carrying *rd* mutation) [1] were used for analysis of G protein expression. Heterozygous *Opn4* ^+*/*−^(*tau*-*LacZ* ^+*/*−^) mice (mixed C57BL/6 and 129/SvJ background) that express a β-gal reporter selectively in M1 type pRGCs [40] were used for localisation of G protein subunits within M1 type pRGCs. *Opn4.Cre* ^+*/*−^ *.EYFP* ^+/+^ mice (mixed C57BL/6 and 129/SvJ background) that express EYFP in all classes of pRGC [10, 41] were used for localisation of G protein subunits within M1-M5 type pRGCs. *Opn4.Cre* ^+*/*−^ *.EYFP* ^+/+^ mice were generated by breeding *Opn4* ^−*/*−^ *.Cre* ^+*/*+^ mice [10] with mice containing a lox.STOP.lox EYFP sequence inserted within the *Rosa26* locus [58], and have been characterised previously [41]. Degenerate *rd/rd cl* mice (90–135 days) lacking rod and cone photoreceptors [1] were used for in vivo gene silencing and pupillometry studies. All animals were housed under a 12:12 LD cycle with food and water ad libitum. All procedures were conducted in accordance with the Animals (Scientific Procedures) Act 1986 and the University of Oxford Policy on the Use of Animals in Scientific Research (PPL 70/6382 and 30/2812).

### Immunohistochemistry

Collection, fixation, cryoprotection and cryostat sectioning of whole mouse eyes were performed as described previously [20]. Immunostaining was performed using standard techniques. Briefly, 18 µm sections were permeabilised in PBS with 0.2 % Triton-X for 20 min at RT then blocked in PBS with 10 % donkey serum (Sigma) for 1 h at RT. Primary antibodies were diluted in PBS with 2.5 % donkey serum and 0.2 % Triton X and incubated for 16 h at 4 °C. Rabbit polyclonal anti-Gnaq/11 1:500 (ab79337, Abcam, QLNLKEYNLV), goat polyclonal anti-Gnaq 1:500 (ab128060, Abcam, NDLDRVADPSYLPT), rabbit polyclonal anti-Gna14 1:1000 (3A-195, Gramsch Labs, QLNLREFNLV), rabbit polyclonal anti-Gβ (sc-378, Santa Cruz Biotech), chicken polyclonal anti-β-Gal 1:1000 (ab9361, Abcam), chicken anti-GFP/EYFP 1:1000 (GFP-1020, AVES Labs), and rabbit polyclonal anti-melanopsin 1:2500 (UF006, Advanced Targeting Systems). Secondary antibodies were donkey anti-rabbit Alexa 568, donkey anti-goat Alexa 633 (Life Technologies) and donkey anti-chicken Alexa 488 (Jackson Immunoresearch) diluted 1:200 in PBS with 2.5 % donkey serum and 0.2 % Triton-X incubated for 2 h at RT. All wash steps were performed with PBS 0.05 % Tween. Sections were mounted with Prolong Gold anti-fade media containing DAPI (Life Technologies). Labelling of melanopsin in whole retina flatmounts was performed as described previously [45]. Images were acquired using a LSM710 laser scanning confocal microscope (Zeiss) and Zen 2009 image acquisition software (Zeiss). Laser lines for excitation were 405, 488, 561 and 633 nm. Emissions were collected between 440–480, 505–550, 580–625 and 650–700 nm for blue, green, red and far red fluorescence respectively. Individual channels were collected sequentially. For all images, global enhancement of brightness and contrast was performed using Zen Lite 2011 software (Zeiss).

### Cell culture and generation of Opn4L and Opn4S stable cell lines

Neuro-2A cells (ECACC) were cultured in DMEM (Sigma) supplemented with 10 % FBS (Life Technologies), 2 mM l-glutamine (Sigma), 1 % (v/v) penicillin/streptomycin (Sigma) in a humidified chamber at 37 °C with 5 % CO_2_. Cells were fed fresh media every 2–3 days and passaged prior to reaching confluence. Stable Neuro-2A cell lines expressing either Opn4L or Opn4S were generated using standard techniques. Briefly, cells were transfected with Opn4L and Opn4S in pIRES2-AcGFP expression vectors (BD Biosciences) as described previously [19]. Stable cell lines were then selected by addition of 800 µg/ml G418 antibiotic (Life Technologies) to the media for 4 weeks and then seeded as single cell cultures to generate clonal cell lines. Levels of melanopsin expression in the resulting cell lines were confirmed via qPCR analysis and western blotting of whole cell lysates using isoforms specific antibodies as described previously [19]. Functional expression of melanopsin was confirmed via whole cell patch clamp recording of melanopsin light activated currents as described previously [19, 33] (Supplementary Fig. 3).

### Time lapse calcium imaging

Monitoring of intracellular calcium levels in Neuro-2A cells was performed using the cell permeant calcium sensitive indicator Rhod-2 AM (Life Technologies) at a final concentration of 5 mM, with 0.1 % DMSO, 0.02 % Pluronic F-127 and 10 mM Probenecid (Life Technologies) incubated for 30 min at 37 °C. Following dye loading cells were washed briefly and incubated for a further 15 min in media containing 20 µM 9-*cis* retinal (Sigma). Calcium imaging was performed in Hanks buffered saline solution (HBSS) without CaCl_2_ (Gibco). All steps were performed under dim red light conditions (>600 nm). Calcium imaging was performed using an inverted Olympus IX71 microscope fitted with a Xenon arc light source with a slit monochromator (Cairn Optoscan). Images were collected via a high sensitivity CCD camera (Cascade 512B, Photometrics) and acquired and analysed using Metafluor imaging software (Molecular Devices). Visualisation and focusing of cell cultures prior to imaging was performed briefly under dim red light by placing a 610 nm band pass filter (Chroma) in the white light path. Images were collected every 2 s using a 100 ms exposure of 545 nm light (bandwidth 10 nm, intensity 7.9 × 10^13^ photons cm^−2^ s^−1^) to excite the Rhod-2 dye, with emitted fluorescence collected via a 610 nm band pass filter (Chroma). Intensity of light stimuli was measured using a radiometrically calibrated spectrophotometer (Ocean Optics, UK). Analysis of changes in Rhod-2 fluorescence was performed by defining regions of interest around individual cells using Metafluor software with data points for individual cells exported to MS Excel for detailed analysis.

### In vitro optimisation of siRNA

siRNA used for in vitro studies were 25mer duplexes with chemical modification to provide increased serum stability and reduced off target effects (Stealth siRNA, Life Technologies). All sequences were designed using the manufacturer’s proprietary algorithm. For each gene of interest, three different siRNA sequences were screened via qPCR for silencing efficiency compared to a non-sequence control (NSC) siRNA not targeting any known gene. siRNA was transfected into Neuro-2A cells using the Lipofectamine RNAi Max transfection reagent (Life Technologies) using the recommended protocols with a final siRNA concentration of 50 nM. Levels of gene silencing were assessed 48 h post transfection by qPCR. For co-transfection of plasmid DNA and siRNA, cells were simultaneously transfected with siRNA (as above) and plasmid DNA (2 µg/35 mm dish) using the Genejuice transfection reagent (Merck) using standard protocols [19]. Sequences of siRNAs selected for functional silencing experiments were as follows; NSC ccuacgccaccaauuucgu, Opn4 ucgcacugauugucauucuucucuu, Gnaq cgcuuagcgaauaugaucaaguucu, Gna11 ccugguuccagaacucgucugucau. Full length coding sequence for mouse Gna14 in the Sport6.1 mammalian expression vector (Life technologies) was obtained from Geneservices (Source Bioscience). Plasmids containing full coding sequences for Gnaq and Gna11 were a kind gift from Wayne Davies, University of Oxford. Plasmid amplification and purification was performed using standard techniques.

### qPCR

Isolation of total RNA from Neuro-2A cells and whole retina was performed using Trizol (Life Technologies) and RNeasy spin columns (Qiagen) using standard protocols [20]. 1 µg cDNA was reverse transcribed into cDNA using Superscript III (Life Technologies) with oligo dT primers (Life Technologies). Quantitative PCR was performed using Sybr Green mastermix on a StepOne thermal cycler (Applied Biosystems, USA) as described previously [19]. Relative quantification of transcript levels was performed as described previously [59]. Primer sequences were as follows (For/Rev); *Opn4* TCACAGGGATGCTGGGCAATC, TTCTTGTAGAGGCTGCTGGCAAAG, *Gnaq* GCTGACCTAAGACCCAAAGG, TATTCCAACCCCAGGTCAAA, *Gna11* TCCTGCACTCACACTTGGTC, AGGATGGTGTCCTTCACAGC, *Gna14* CAGGTTCTGGCTGAGTGTGA, TCAGAAACCAGGGGTAGGTG. Levels of target gene expression were normalised to the geometric mean expression of three house-keeping genes [60], *Arp, Gapdh* and *β*-*actin* using primer sequences reported previously [61].

### In vivo delivery of siRNA

For in vivo intravitreal injections, HPLC in vivo grade siRNA was obtained from Dharmacon (Thermo Scientific) based on the sequences validated in vitro, but also included specific backbone modifications (siSTABLE formulation) to reduce off target effects and increase stability under in vivo conditions (Dharmacon propriety information). 1 µg of siRNA was complexed to Invivofectamine 2.0 transfection reagent (Life Technologies) using the manufacturer’s recommended protocols, with a final injection volume of 1.5 µl per eye (in saline with 4 % glucose). Intravitreal injections were performed on ketamine-meditomidine anaesthetised animals using a 34 gauge needle (point style 2) with a 5 μl Hamilton syringe. For each animal siRNA targeting a gene of interest was injected into one eye with the opposing eye receiving injections of NSC siRNA.

### Pupillometry

Assessment of pupillary light responses was performed 48–72 h post intravitreal injection of siRNA, using methods reported previously [45]. Testing was performed between ZT 4–8, and all animals were dark adapted for 1–2 h prior to testing. Five minutes prior to recording, a 1 % tropicamide was applied to the stimulated eye. A xenon arc lamp (150 W solar simulator, Lot Oriel, UK) with a 480 nm monochromatic filter (Andover, 10 nm half-bandwidth) was used to produce a light intensity of 14.6 log quanta/cm^2^/s (173 μW/cm^2^/s). Irradiance measurements were made using a radiometrically calibrated spectrophotometer (Ocean Optics, UK). Light stimuli (10 s) were transmitted to the eye via a liquid light guide as an irradiant light stimulus using a 2″ integrating sphere (Pro-lite Technology, UK) and was controlled by a shutter positioned in the light path (LSZ160 shutter, Lot Oriel UK; custom software supplied by BRSL, Newbury, UK). Images of consensual pupil responses were collected with a Prosilica NIR sensitive CCD video camera (BRSL, Newbury, UK) at a rate of 10 frames per second, under infra red LED illumination (850 nm, 10 nm half-bandwidth). During pupil measurements unanaesthetised animals were temporarily restrained using normal husbandry techniques for the duration of the recording (29 s, including baseline, stimulation and recovery phases). Each animal was tested on multiple occasions to minimise any artefacts due to handling. Data reported for each individual animal represent the mean of all individual trials. All images were analysed using ImageJ software (NIH; rsbweb.nih.gov/ij/).

### Statistical analysis

All data are shown as mean ± SEM. Statistical analysis was performed using unpaired two-tailed Student’s *t* test. Where stated Bonferroni multiple test corrections were applied.

## Electronic supplementary material

Below is the link to the electronic supplementary material.

Supplementary material 1 (PDF 8461 kb)
