# Supplementary material for: Using siRNA to define functional interactions between melanopsin and multiple G Protein partners
Source: Cell Mol Life Sci. 2014 Jun 24;72(1):165–79. doi: 10.1007/s00018-014-1664-6 (PMC4282707; doi:10.1007/s00018-014-1664-6)
Supplement: Supplementary file 1 — Supplementary material 1 (PDF 8461 kb) [file 18_2014_1664_MOESM1_ESM.pdf]

Supplementary Material
Supplementary Figure 1

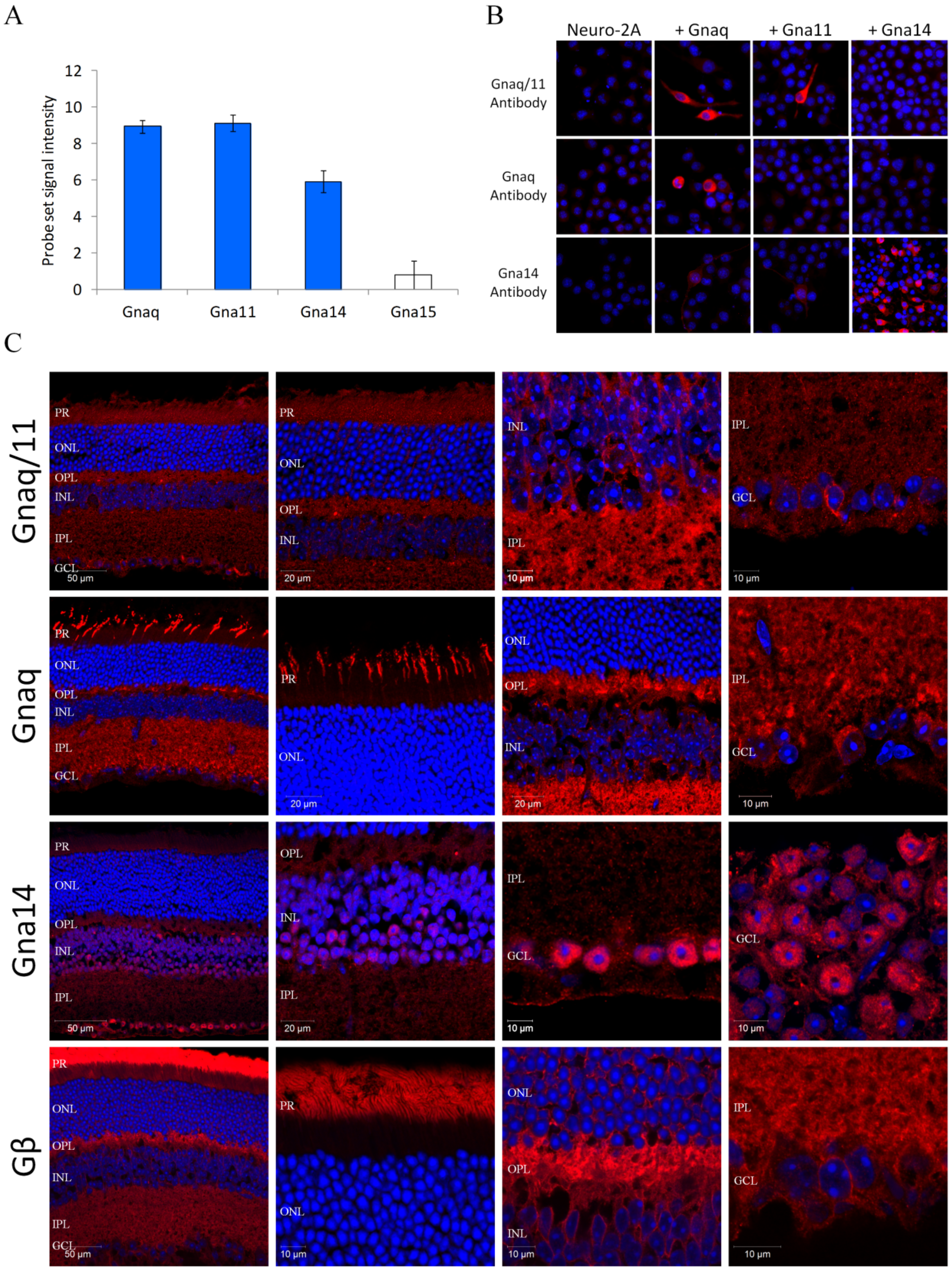

Supplementary Figure 1. Expression of Gnaq/11 type G-proteins in the mouse retina. A) Gene array analysis was performed using GeneChip Mouse Exon 1.0 ST Arrays (Affymetrix), using methods described previously [1]. Gene microarray analysis confirms the expression of Gnaq, Gna11 and Gna14 in the adult mouse retina, with expression of Gna15

not detected. Values shown are the mean of array signal values of all probe sets for each gene of interest. Probe sets not showing significant p-values (<0.05) were given a value of 0. The number of probe sets returning significant values was as follows; Gnaq, 10 of 10 probe sets, p-values ranging from 0.01 to 4.6E-09; Gna11, 7 of 7, p-values ranging from 0.004 to 6.3E-08; Gna14, 12 of 12, p-values ranging from 0.03 to 2.6E-06; Gna15 1 of 8 probe sets, p=0.003. Expression of Gna15 was deemed to be absent as 7 of 8 probe sets failed to return significant signal values. **B)** The specificity of Gnaq/11, Gnaq and Gna14 antibodies was determined by their ability to successfully label Neuro-2A cells transfected with their target proteins. The Gnaq/11 antibody brightly labelled cells transfected with Gnaq and Gna11, but not Gna14. The Gnaq antibody was specific for Gnaq. The Gna14 antibody brightly labelled Gna14 transfected cells, but due to a high sequence homology (and high levels of protein expression in this system) a low level of staining was occasionally also observed for Gnaq and Gna11 transfected cells. Staining was absent from wildtype untransfected cells following incubation with all antibodies. **C)** Images showing the localisation of Gnaq/11 type G-proteins in the mouse retina as shown by labelling with antibodies that recognise both Gnaq and Gna11, termed Gnaq//11, or antibodies specific for Gnaq, Gna14 or Gβ subunits. Note the similarities in pattern of staining observed for Gnaq/11, Gnaq and Gβ antibodies. For all images DAPI nuclear counter stain is shown in blue. PR, Photoreceptors; ONL, outer nuclear layer; OPL, outer plexiform layer; INL, inner nuclear layer; IPL, inner plexiform layer; GCL, ganglion cell layer.

Supplementary Figure 2

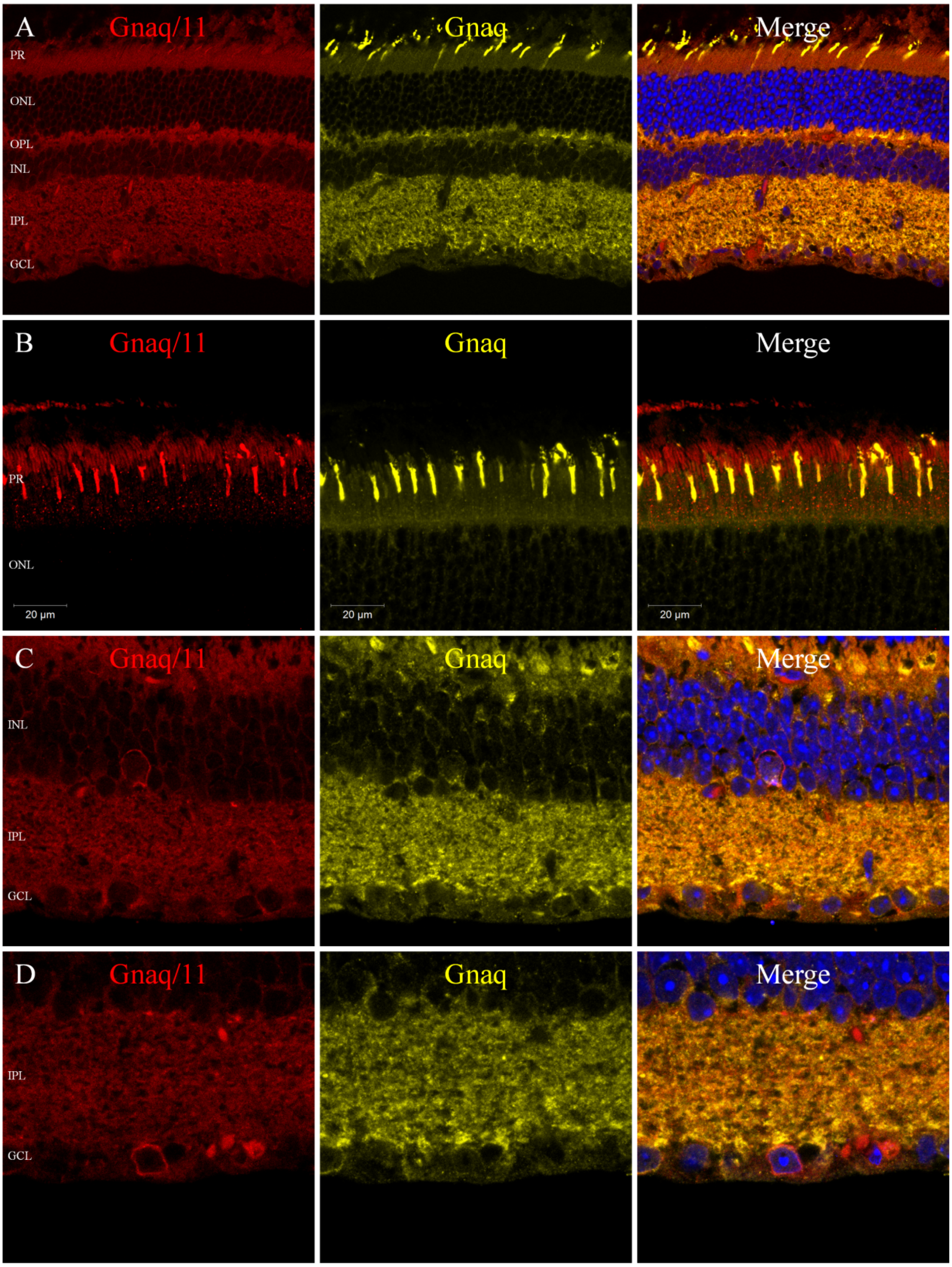

Supplementary Figure 2. Comparison of Gnaq/11 and Gnaq antibodies reveals a distinctive pattern of Gna11 expression in the mouse retina. Overall patterns of Gnaq/11 and Gnaq labelling are highly similar (**A**), yet only Gna11 is seemingly detected in rods, with high levels of Gnaq observed in cone photoreceptors (**B**). In both the ganglion cell layer and more rarely in the inner nuclear layer cells are identified that show distinctive labelling for Gnaq/11

but show not significant increase in Gnaq labelling (**C-D**). We conclude this likely represents increased levels of Gna11 expression within these cells. For all images DAPI nuclear counter stain is shown in blue. PR, Photoreceptrs; ONL, outer nuclear layer; OPL, outer plexiform layer; INL, inner nuclear layer; IPL, inner plexiform layer; GCL, ganglion cell layer.

Supplementary Figure 3

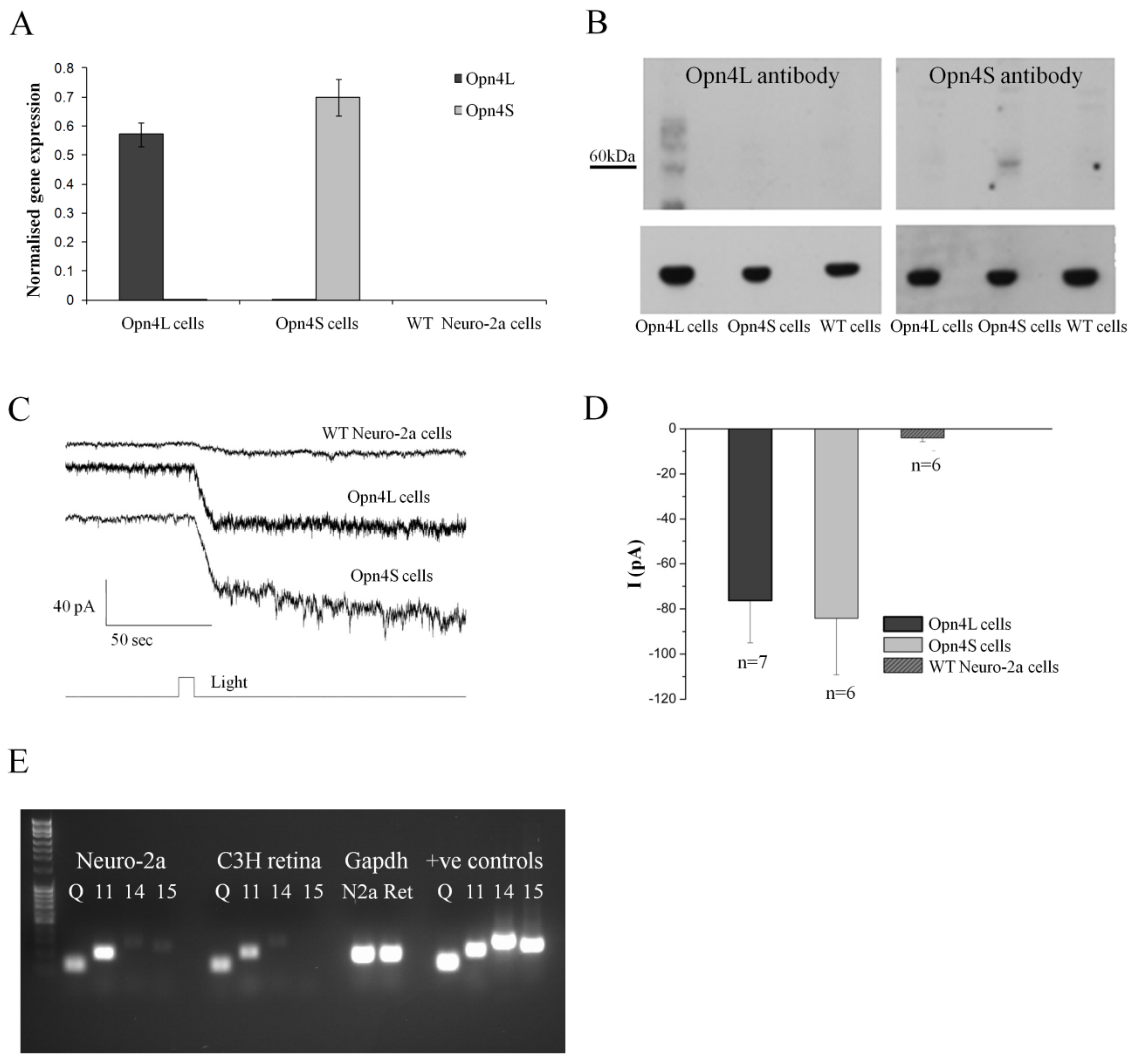

Supplementary Figure 3. Characterisation of melanopsin expressing stable cell lines. **A**) qPCR analysis confirming levels of Opn4L and Opn4S expression in the respective cell lines. No expression of Opn4L or Opn4S was detected in the opposing cell lines and neither Opn4L nor Opn4S were detected in wildtype (WT) Neuro-2A cells. Levels of gene expression are shown normalised to the geometric mean of 3 housekeeping genes, (Arp, Gapdh, β-actin). **B**) Western blotting of whole cell lysates from Opn4L, Opn4S and WT Neuro-2A cells using Opn4-Long and Opn4-Short isoform specific antibodies [2] (upper panels) and β-actin (lower panels) shows that expression of Opn4L and Opn4S protein are detected in the respective cell lines, with immunoreactive proteins detected at ~55 and

60kDa by the short and long isoform specific antibodies respectively. These masses are comparable to the calculated molecular weights of 51kDa for Opn4S and 57kDa for Opn4L (Pires et al., 2009). **C**) Whole cell patch clamp recordings of light induced currents observed in Opn4L and Opn4S expressing Neuro-2A cells and WT Neuro-2A cells following stimulation with 420nm light (8 x 10^{14} photons cm^{-2} s^{-1}). Light activated inward currents are observed for melanopsin expressing cells but not WT Neuro-2A cells. Traces are offset for clarity. **D**) Summary of patch clamp data, showing similar amplitudes of response in both Opn4L and Opn4S cell lines. Data is shown as mean ± SEM. The methods used for whole cell recordings of opsin light response are the same as those described previously [2-5]. **E**) PCR analysis shows high levels of Gnaq and Gna11 mRNA expression within Neuro-2A cells and whole retina samples. Low levels of Gna14 and Gna15 mRNA were also detected within Neuro-2A cells, and low levels of Gna14 but not Gna15 were detected in whole retina samples. PCR products for Gapdh are shown as a template loading control. PCR reactions using plasmids containing the relevant G protein coding sequences as template DNA were used as positive controls.

Supplementary Figure 4

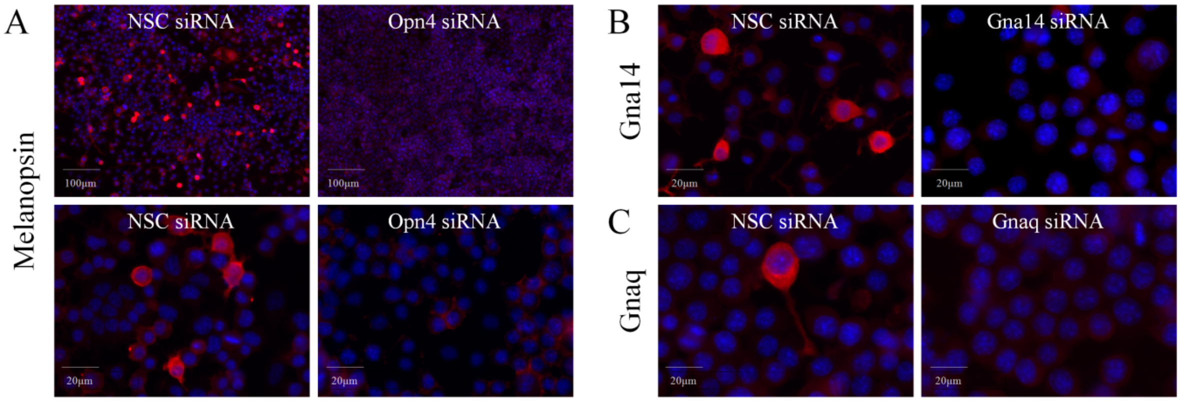

Supplementary Figure 4. Levels of protein silencing observed for Neuro-2A cells transiently transfected with plasmid DNA encoding melanopsin (A), Gna14 (B) and Gnaq (C) and corresponding siRNA or NSC control siRNA. Levels of protein silencing were determined by immunostaining with melanopsin, Gnaq and Gna14 antibodies respectively. Note that only minimal levels of target proteins are detected 48 hours after delivery of Opn4, Gnaq and Gna14 siRNA. DAPI nuclear counter stain is shown in blue.

Supplementary Figure 5

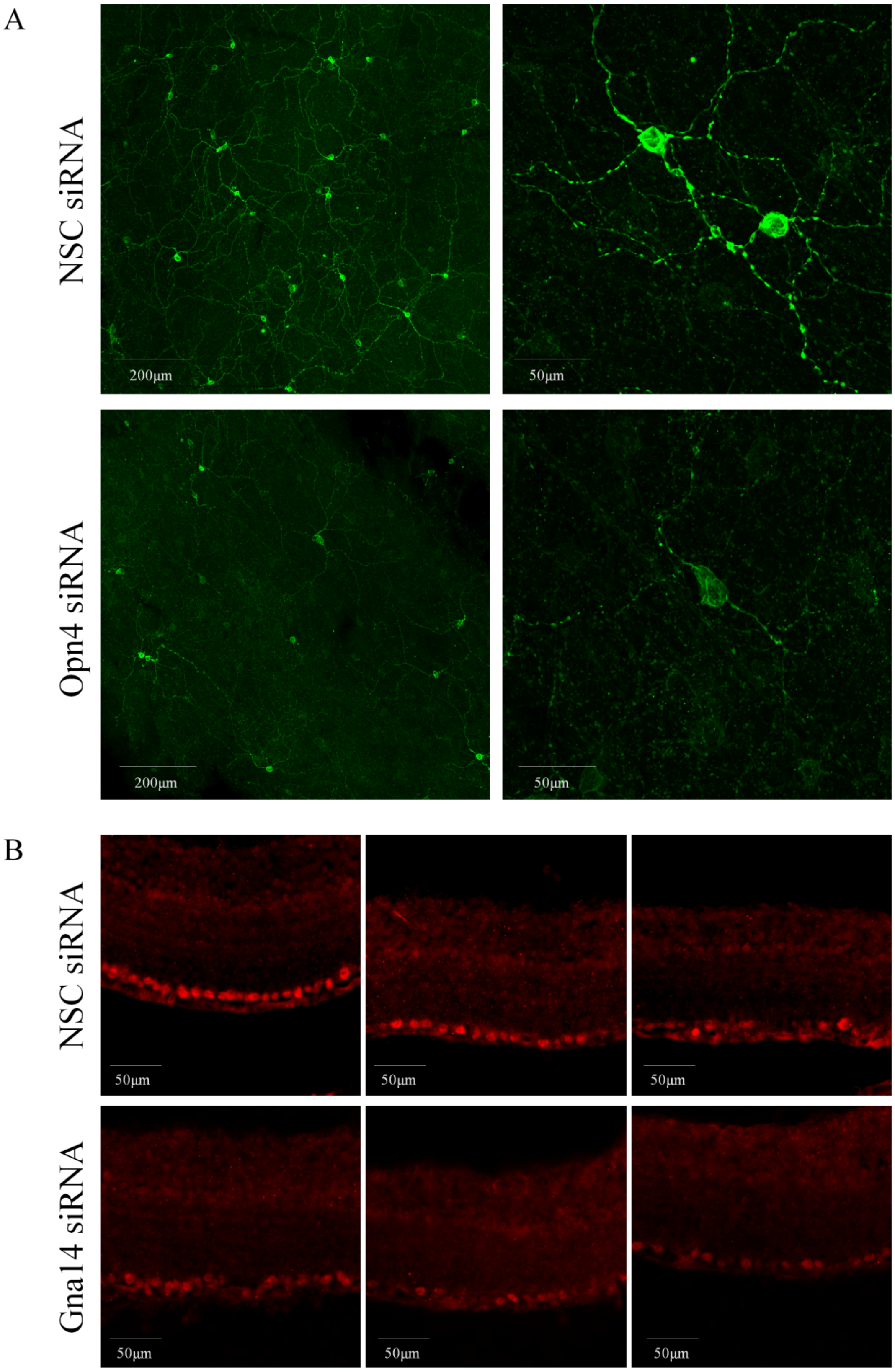

Supplementary Figure 5. Images showing levels of protein silencing detected following *in vivo* administration of siRNA. Images shown are enlarged versions of the images shown in Figure 5 of the main manuscript.

Supplementary References

1. Jagannath, A., Butler, R., Godinho, S.I., Couch, Y., Brown, L.A., Vasudevan, S.R., Flanagan, K.C., Anthony, D., Churchill, G.C., Wood, M.J., et al. (2013). The CRTC1-SIK1 pathway regulates entrainment of the circadian clock. Cell *154*, 1100-1111.
2. Pires, S.S., Hughes, S., Turton, M., Melyan, Z., Peirson, S.N., Zheng, L., Kosmaoglou, M., Bellingham, J., Cheetham, M.E., Lucas, R.J., et al. (2009). Differential expression of two distinct functional isoforms of melanopsin (Opn4) in the mammalian retina. J Neurosci *29*, 12332-12342.
3. Melyan, Z., Tarttelin, E.E., Bellingham, J., Lucas, R.J., and Hankins, M.W. (2005). Addition of human melanopsin renders mammalian cells photoresponsive. Nature *433*, 741-745.
4. Davies, W.I., Zheng, L., Hughes, S., Tamai, T.K., Turton, M., Halford, S., Foster, R.G., Whitmore, D., and Hankins, M.W. (2011). Functional diversity of melanopsins and their global expression in the teleost retina. Cell Mol Life Sci *68*, 4115-4132.
5. Halford, S., Pires, S.S., Turton, M., Zheng, L., Gonzalez-Menendez, I., Davies, W.L., Peirson, S.N., Garcia-Fernandez, J.M., Hankins, M.W., and Foster, R.G. (2009). VA opsin-based photoreceptors in the hypothalamus of birds. Curr Biol *19*, 1396-1402.
